# The modulation of platelet function by growth hormone in growth hormone deficient Hypopituitary patients

**DOI:** 10.1186/s12902-023-01448-6

**Published:** 2023-09-14

**Authors:** Irene K Oglesby, David Slattery, Nigel Glynn, Saket Gupta, Karen Duggan, Martin Cuesta, Eimear Dunne, Aoife Garrahy, Siobhan Toner, Dermot Kenny, Amar Agha

**Affiliations:** 1 Irish Centre for Vascular Biology and Molecular and Cellular Therapeutics, Royal College of Surgeons in Ireland, Dublin, Ireland; 2 Department of Medicine, Royal College of Surgeons in Ireland, Dublin, Ireland; 3 Department of Endocrinology, Beaumont Hospital, Dublin 9, Dublin, Ireland

**Keywords:** Hypopituitarism, Growth hormone, Platelets, von Willebrand factor, Thrombosis, Platelet-vWF interaction

## Abstract

**Background:**

Growth hormone deficiency (GHD) has been implicated in increased cardiovascular and cerebrovascular disease risk seen in hypopituitarism, however the mechanism remains speculative. We hypothesise that platelet abnormalities may play a contributory role. Herein we examined platelet behaviour in GHD hypopituitary patients, pre- and post-growth hormone (GH) replacement.

**Methods:**

This study utilizes a physiological flow-based assay to quantify platelet function in whole blood from patient cohorts under arterial shear. Thirteen GH Naïve hypopituitary adults with GHD and thirteen healthy matched controls were studied. Patients were assessed before and after GH treatment. All other pituitary replacements were optimised before the study. In addition to a full endocrine profile, whole blood was labelled and perfused over immobilised von Willibrand factor (vWF). Seven parameters of dynamic platelet-vWF interactions were recorded using digital image microscopy and analysed by customised platelet tracking software.

**Results:**

We found a significantly altered profile of platelet-vWF interactions in GHD individuals compared to healthy controls. Specifically, we observed a marked increase in platelets shown to form associations such as tethering, rolling and adherence to immobilized vWF, which were reduced post GH treatment. Speed and distance platelets travelled across vWF was similar between controls and pre-therapy GHD patients, however, this was considerably increased post treatment. This may indicate reduced platelet signaling resulting in less stable adhesion of platelets post GH treatment.

**Conclusions:**

Taken together observed differences in platelet behaviour may contribute to an increased risk of thrombosis in GHD which can in part be reversed by GH therapy.

## Introduction

Several observational studies have shown that people with hypopituitarism have a reduced life expectancy when compared to the background population [1–3]. A pioneering study by Rosen and Bengtsson demonstrated an overall standardised mortality ratio (SMR) of 1.81 for those diagnosed with hypopituitarism, 1.47 vs. 2.82 for males and females respectively. The main cause of mortality in this study was vascular disease, with an SMR in the overall group of 1.95, (1.7 vs. 2.7 M:F) [1]. Several other studies supported this initial finding by demonstrating increased vascular mortality [3]. Another study by Tomlinson et al., attributed the main cause of mortality to cerebrovascular disease (SMR 2.55) with cardiovascular disease having an SMR of 1.62 [2]. The reason behind this higher rate of vascular disease is unclear and may be multifactorial [3]. Growth hormone deficiency (GHD), is the most common deficiency seen in pituitary disease [4] and while unproven, is speculated to be a contributing factor towards the pathogenesis of vascular disease in this cohort [2, 5]. A favourable effect of growth hormone therapy (GHT) on several vascular disease risk factors has been reported in a number of studies including lipid profile, [6, 7] central adiposity/body composition, [8–10] carotid artery intima thickness [11, 12] and endothelial cell function [13]. However, no long-term interventional studies examined the effect of growth hormone therapy on hard end points such as vascular morbidity and mortality.

Platelets play a significant role in the pathogenesis of vascular disease [14, 15] as a direct result of their contribution to the haemostatic process. Thrombus formation as a result of platelet exposure to ruptured atheromatous plaques in the endothelium can lead to coronary, cerebral and peripheral vascular disease [16–20]. These events are initiated via the association of platelets with exposed matrix proteins such as von Willebrand factor (vWF) at the site of vessel injury. Initial platelet-vWF interactions are short lived where both tethering and rolling (translocation) of platelets along the vessel surface is mediated by on-off binding to the glycoprotein (GP)Ibα receptor. A complex signalling cascade is then initiated, culminating in the activation of the integrin GPIIb/IIIa receptor causing cross-linkage, subsequent stable adhesion of platelets to vWF and ultimately thrombosis [21, 22].

In this exploratory study we employed a well characterised physiological flow-based assay that quantifies platelet function in whole blood under arterial shear in order to study platelet behaviour in a small cohort of GHD hypopituitary patients at baseline and at 3 months post growth hormone replacement in comparison to healthy control individuals.

## Materials and methods

### Patients and study design

This was a prospective interventional exploratory study of thirteen randomly selected hypopituitary patients (7 males) with GHD recruited from the pituitary clinic in Beaumont Hospital, along with 13 healthy, matched controls (Table 1). All patients were adults who had organic pituitary disease with biochemical confirmation of severe GHD by an insulin tolerance test (ITT) using the GH Research Society Criteria. Other pituitary deficits were diagnosed according to standard criteria described elsewhere [23]. Exclusion criteria for both groups included: pregnancy, a history of malignancy, diabetes mellitus, heart failure, chronic kidney disease, chronic liver disease and disorders of platelet function and blood clotting.

Non–functioning pituitary adenomas (NFPAs) was the commonest group in the GHD patient cohort accounting for eight of the 13 cases. This was followed by craniopharyngiomas in two subjects, and the remaining three with Neurosarcoidosis, Prolactinoma and TSHoma (normal Thyroid function post treatment). 12 out of 13 patients underwent pituitary surgery with four also receiving radiation therapy. In addition to GHT for all 13 participants, the number of individuals receiving hydrocortisone, thyroxine, Oestrogen and Testosterone therapy were 8, 6, 3 and 5 respectively. Importantly, one patient was taking aspirin 75 mg daily. The platelet translocation profile of this patient did not appear to differ significantly from the rest however this data and that of the matched healthy control was excluded in final analysis.

Anthropometric measurements including blood pressure, weight, height and waist circumference were recorded, along with tumour type/disease resulting in hypopituitarism and treatment received. All pituitary hormone replacement therapy other than GH was optimised for at least 3 months before the start of the study. Full endocrine and metabolic profiles were measured. Whole blood was drawn for platelet function assessment. GHD patients were treated with recombinant growth hormone (Genotropin, Pfizer Endocrine Care), with the dose adjusted every 2 weeks to achieve a target insulin like growth factor – 1 (IGF-1) level in the upper third of the normal reference range. Platelet function was assessed in patients before and 3 months after achieving a stable maintenance dose of growth GHT. The Healthy control subjects were studied on one occasion only.

The Beaumont Hospital ethics committee approved this study and all subjects gave written, informed consent.

### Assessment of platelet function

In order to elucidate the role platelets play in the pathogenesis of ischemic syndromes it is critical to be able to measure their dynamic behaviour in an environment that can mimic in vivo flow and shear. We have previously described a dynamic platelet function assay (DPFA) [24] capable of detecting subtle differences in platelet function in different age groups, term versus pre-term neonates, pregnancy and pre vs. post kidney transplant [25–27]. This assay measures platelet function in microliter amounts of whole blood utilizing parallel plate flow chambers coated with immobilized vWF under arterial shear conditions. DPFA outputs relating to distinct biological platelet functions are measured using custom-designed platelet tracking software [28]. Resulting platelet measurements provide an insight into the extent of platelet population interactions with vWF.

#### Preparation of blood samples

Venous blood was collected from the antecubital vein using a 20-gauge butterfly needle connected to a citrated Sarstedt Monovette syringe (Drinagh Co. Wexford, Ireland). Blood samples were kept at room temperature with gentle rocking and used within 1 h of phlebotomy.

#### Dynamic platelet function assay (DPFA)

The Dynamic Platelet Function Assay was performed as previously described [24–27]. Briefly, custom parallel plate perfusion chambers were coated overnight with 100 µg/ml vWF, washed with PBS and blocked with 1% BSA for one hour prior to use. Whole blood was labelled with 1 μm DiOC6 fluorescent dye for five minutes at 37ºC prior to perfusion through the chamber at an arterial rate of shear (1500 s-1).

Platelet translocation behaviour was recorded using real-time video microscopy where the key output from this procedure is a time-sequenced set of grayscale images or frames, captured at a rate of 30 frames per second (fps), of a 133 × 133 µm [2] section (field of view) of the surface. Image stacks capturing platelet interactions were analysed by a custom designed and validated software package [28]. Only those platelets that interact with the vWF surface are captured by the imaging software (Metamorph® Image Analysis Software, Molecular Devices, LLC, Sunnyvale, CA, USA). This assay monitors the early stages of platelet adhesion and interaction over 16.7 s of each flow run experiment, prior to the occurrence of largescale platelet aggregation and thrombus formation.

Seven parameters relating to various aspects of dynamic platelet behaviour were obtained from the analysis as described by Ralph et al., 2016 [28] and employed to assess the following traits: **tethering** to the vWF surface (number of platelet tracks), **rolling** on the surface after initial vWF interaction (number of translocating platelets, translocation distance and speed) and **adherence to vWF** (stably adhered platelets, adhesion rate and percentage of surface covered by platelets).

### Statistical analysis

Statistical analysis was performed using non-parametric tests; The Wilcoxon matched-pairs signed-rank test, a non-parametric method to compare before-after, or matched subjects for GHD pre and post GHT analysis and the Mann Whitney U-test, two tailed p value or one way ANOVA (Multiple comparisons) where appropriate, when comparing both GHD cohorts and healthy controls. Statistical analysis of anthropometric data was performed using SPSS version 21.0. Baseline characteristics are presented as median and interquartile range. All platelet data analyses were performed using GraphPad PRISM 8.4.3 software package (San Diego, CA). All blood samples were run in triplicate in the DPFA and the mean value was determined for each measured parameter obtained from platelet interaction analysis. Platelet parameter analyses are expressed as the mean ± SD. Values were considered statistically significant where p < 0.05.

## Results

### Study population characteristics

#### GHD and healthy control subjects – baseline characteristics

The baseline characteristics of both the GHD and control groups can be seen in Table 1. Both groups were matched for age and sex. IGF-1 was significantly higher in controls as expected while white cell count (WCC) was significantly higher in the GHD group.

**Table 1:** Baseline characteristics of GHD subjects and controls. Values are displayed as median and (interquartile range)

Measurement	GHD (n = 13, M = 7)	Controls (n = 13, M = 7)	P value
Age(years)	47(38.5–55.5)	52(43.5–64.5)	0.2
IGF-1(ng/ml)	127.8(101.9-132.25)	180.7(171.8–196)	0.004^**^
Free T4(pmol/l)	9.3(8.15–12.35)	10.7(9.95–11.95)	0.42
Haemaglobin(g/dL)	13.8(12.2-14.15)	13.5(12.6–15.8)	0.31
Platelet count(10^9^ /L)	261(222–282)	234(194–305)	0.59
WCC(10^9^ /L)	6.97(5.43–8.92)	4.48(3.85–7.58)	0.04^*^
INR	1.0(0.96-1.0)	1.05(1.0-1.08)	0.57
Creatinine(umol/l)	75(62–83)	69(52.5–74)	0.18
Total cholesterol(mmol/l)	5.39(4.48–6.76)	5.44(3.56–5.78)	0.54
LDL-C(mmol/l)	3.11(2.46–4.63)	2.63(1.32–3.84)	0.29
HDL-C(mmol/l)	1.35(0.98–1.72)	1.37(0.84–1.59)	0.68
Triglycerides(mmol/l)	1.38(1.13–1.58)	0.97(0.85–1.81)	0.2
Fasting glucose(mmol/l)	4.8(4.5–5.25)	5.3(4.85–6.25)	0.06
Testosterone(males,nmol/l)	19.5(16.9–22.5)	16.3(10.2–20.7)	0.25
HbA1C(DCCT)	5.4(5.4–5.6)	5.5(5.15–6.4)	0.59
Fasting Insulin(mIU/L)	5.6(2.9–9.55)	8(3.5-12.25)	0.40
Albumin(g/L)	44(41.25–45.75)	43(39.5–46)	0.69

Insulin-like growth factor I (IGF-1), thyroid-stimulating hormone (TSH), white cell count, (WCC), international normalised ratio (INR), low-density lipoprotein cholesterol (LDL-C), high-density lipoprotein cholesterol (HDL-C), Glycated haemoglobin (HbA1C)

Values are displayed as median (Interquartile range). A Mann Whitney u-test was used to assess for statistically significant differences at baseline between the GHD group and controls. Values were considered statistically significant where p < 0.05*, < 0.01**, < 0.00

#### Clinical findings in GHD subjects

The endocrinological, biochemical and haematological profiles together with anthropometric and BP measurements of the GHD group pre and post GHT are shown in Table 2. IGF-1 levels as expected, were significantly higher post GHT (Table 2). The only other statistically significant difference measured post GHT, was that of fasting insulin levels which were higher after GH treatment indicating early post-therapy insulin resistance induced by GH.

**Table 2:** Measurements and profiles of GHD group pre and post GHRT. Values are displayed as median and (interquartile range)

Measurement	Baseline(n = 13)	Post GHRT(n = 13)	P value
WC(cm)	112(96.5-114.5)	107(98-115.5)	0.26
Systolic BP(mmHg)	115(111.5–127)	121(115.5–134)	0.17
Diastolic BP(mmHg)	80(74-81.5)	79(75-83.5)	0.76
IGF-1(ng/ml)	127.8(101.9-132.25)	181.9(158.1-219.1)	< 0.001***
GH(ng/ml)	0.19(0.07–0.41)	0.29(0.16–0.54)	0.98
TSH(mIU/ml)	1.18(0.81–1.61)	1.16(0.12–1.9)	0.47
T4(pmol/l)	9.3(8.15–12.35)	9(8.15–11.65)	0.32
Haemaglobin(g/dL)	13.8(12.2-14.15)	13.5(12.5–14.7)	1.0
Platelet count(10^9^ /L)	261(222–282)	234(219–272)	0.7
WCC(10^9^ /L)	6.97(5.43–8.92)	6.64(5.29–7.94)	0.33
INR	1.0(0.96-1.0)	0.99(0.94-1.0)	0.38
Creatinine(umol/l)	75(62–83)	69(63-84.5)	0.44
Cholesterol(mmol/l)	5.39(4.48–6.76)	5.61(4.51–6.2)	0.43
LDL(mmol/l)	3.11(2.46–4.63)	2.89(2.61–3.96)	0.55
HDL(mmol/l)	1.35(0.98–1.72)	1.29(0.96–1.57)	0.76
Triglycerides(mmol/l)	1.38(1.13–1.58)	1.35(0.93–1.83)	0.27
Fasting BG(mmol/l)	4.8(4.5–5.25)	4.8(4.4–5.2)	0.71
Testosterone(nmol/l)	19.5(16.9–22.5)	16.1(12.6–21.7)	0.25
Oestrogen females (pg/ml)	95(82–391)	134(77.5-258.5)	0.42
HbA1C(DCCT)	5.4(5.4–5.6)	5.5(5.3–5.6)	0.83
Fasting Insulin(mIU/L)	5.6(2.9–9.55)	6.3(2.8–18.5)	0.04*
Albumin(g/L)	44(41.25–45.75)	42(40-45.5)	0.08

Waist circumference (WC) Insulin-like growth factor I (IGF-1), growth hormone (GH), thyroid-stimulating hormone (TSH), Thyroxine (T4), white cell count, (WCC), international normalised ratio (INR), low-density lipoprotein (LDL), high-density lipoprotein (HDL), Glycated haemoglobin (HbA1C)

Values are displayed as median (Interquartile range). The Wilcoxon matched-pairs signed-rank test was used to compare parameters measured pre and post treatment with GH in the GHD group. Values were considered statistically significant where p < 0.05*, < 0.01**, < 0.001***.

### Platelet reactivity

#### GHD cohort data - pre vs. post GHT (n = 12)

*GHD platelets travel further and faster on vWF following growth hormone replacement therapy*.

**Fig. 1:**
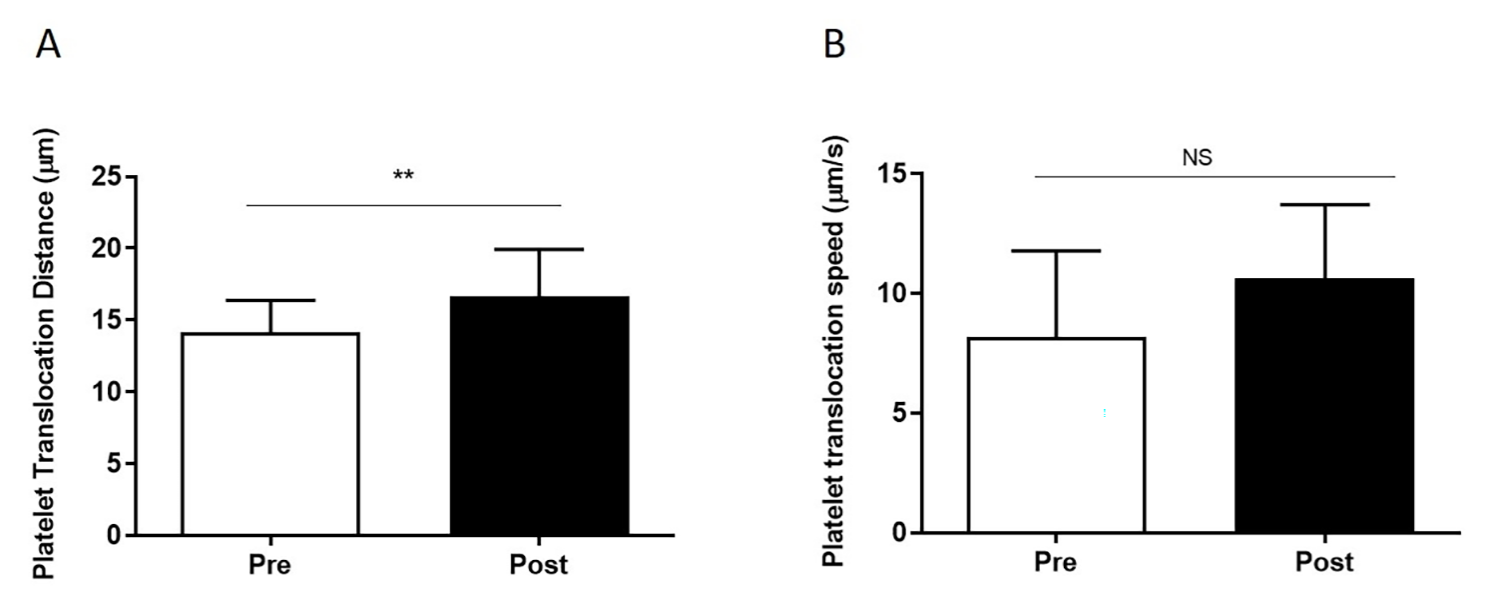
**Platelet-vWF translocation properties in GHD pre vs. post GHT** Platelet translocation distance and speed are increased in GHD subjects post GHT (n = 12 in each group). (**A**) Platelet translocation distance (µm) and (**B**) Platelet translocation speed (µm/s) measured using the dynamic platelet function assay (DPFA). Data are represented as Mean ± SD (*p < 0.05, **p < 0.01, ***p < 0.001). All samples were measured in triplicate

In parameters used to measure **platelet rolling** behaviour i.e. the number of translocating platelets, translocation distance and speed, GHD platelets post GHT replacement were found to travel a significantly further distance (13 ± 0.7 μm vs.17 ± 0.9 μm, pre vs. post, p = 0.005) than pre GHT platelets. Platelet speed (measured in µm/second) post GHT was also increased compared to pre therapy (8.2 ± 1 μm/s vs. 10.8 ± 0.8 μm/s, pre vs. post), however not significantly so, Fig. 1. There were no significant differences observed in platelet translocation numbers in pre vs. post GHT, where both increases and decreases were recorded, (n = 5 and n = 7 respectively). Differences in **platelet tethering** and **adherence** to the immobilized vWF surface also remained largely unchanged following GHT, where seven of the twelve GHD subjects exhibited reduced platelet-vWF interactions post GHT in parameters measuring the number of platelet tracks, stably adhered platelets and adhesion rate and the other 5 subjects displayed increased numbers (data not shown).

#### GHD pre and post GHT vs. healthy controls

*Initial platelet-vWF interactions are increased in GHD groups (pre and post GHT) compared to healthy controls.*

**Fig. 2:**
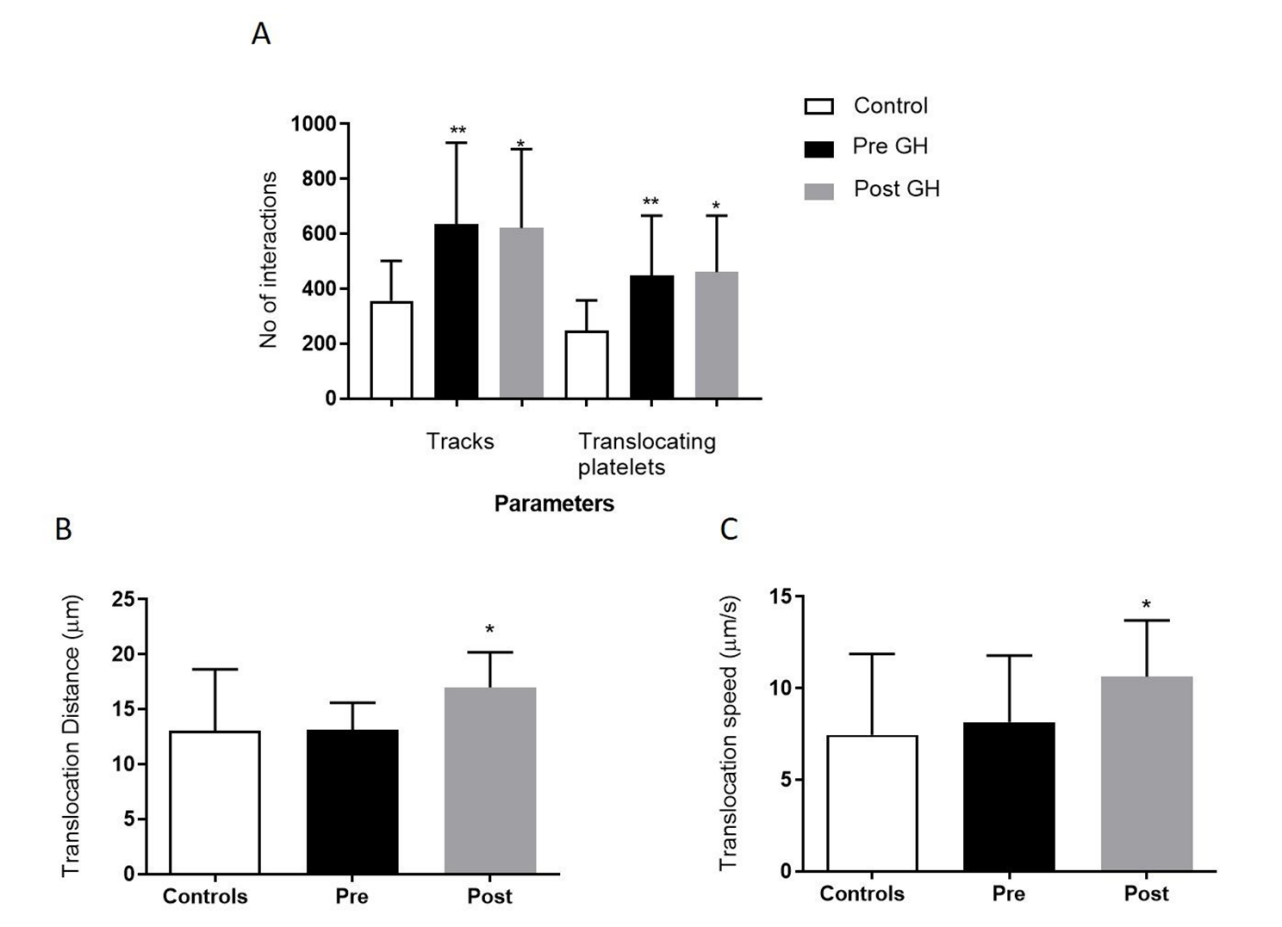
**Platelet –vWF tethering and rolling in GHD pre and post GHT vs. healthy controls** Parameter outputs of platelet tethering and rolling behaviours are increased in GHD subjects, (n = 12) vs. healthy controls (n = 12) both pre- and post GHT. (**A**) Number of platelet tracks and translocating platelets, (**B**) Translocation distance and (**C**) Translocation speed measured using the dynamic platelet function assay. Data are represented as Mean ± SD (*p < 0.05, **p < 0.01, ***p < 0.001). All samples were measured in triplicate in the DPFA

Five of the seven platelet parameters measured were significantly altered when GHD pre and post GHT were compared to healthy controls. **Platelet tethering** to vWF as measured by the number of platelet tracks indicates the overall extent of initial platelet-vWF interactions and saltatory motion mediated by GP(Ib). In comparison to healthy controls there was a significant increase in platelet tracks in the GHD pre therapy cohort (p = 0.001). Post GHT the number of tracks vs. healthy controls was also significantly increased but somewhat reduced compared to pre therapy counts (p = 0.039), Fig. 2A. With respect to platelet **“rolling”** behaviours the number of translocating platelets was found to be significantly higher in both GHD pre and post therapy vs. controls (p = 0.002 and p = 0.02 respectively), Fig. 2A. The speed and distance platelets travelled across vWF were similar between the control group and pre-therapy GHD patients however this was seen to substantially increase post GHT, P = 0.045 and p = 0.03 respectively, Fig. 2B C.

**Fig. 3:**
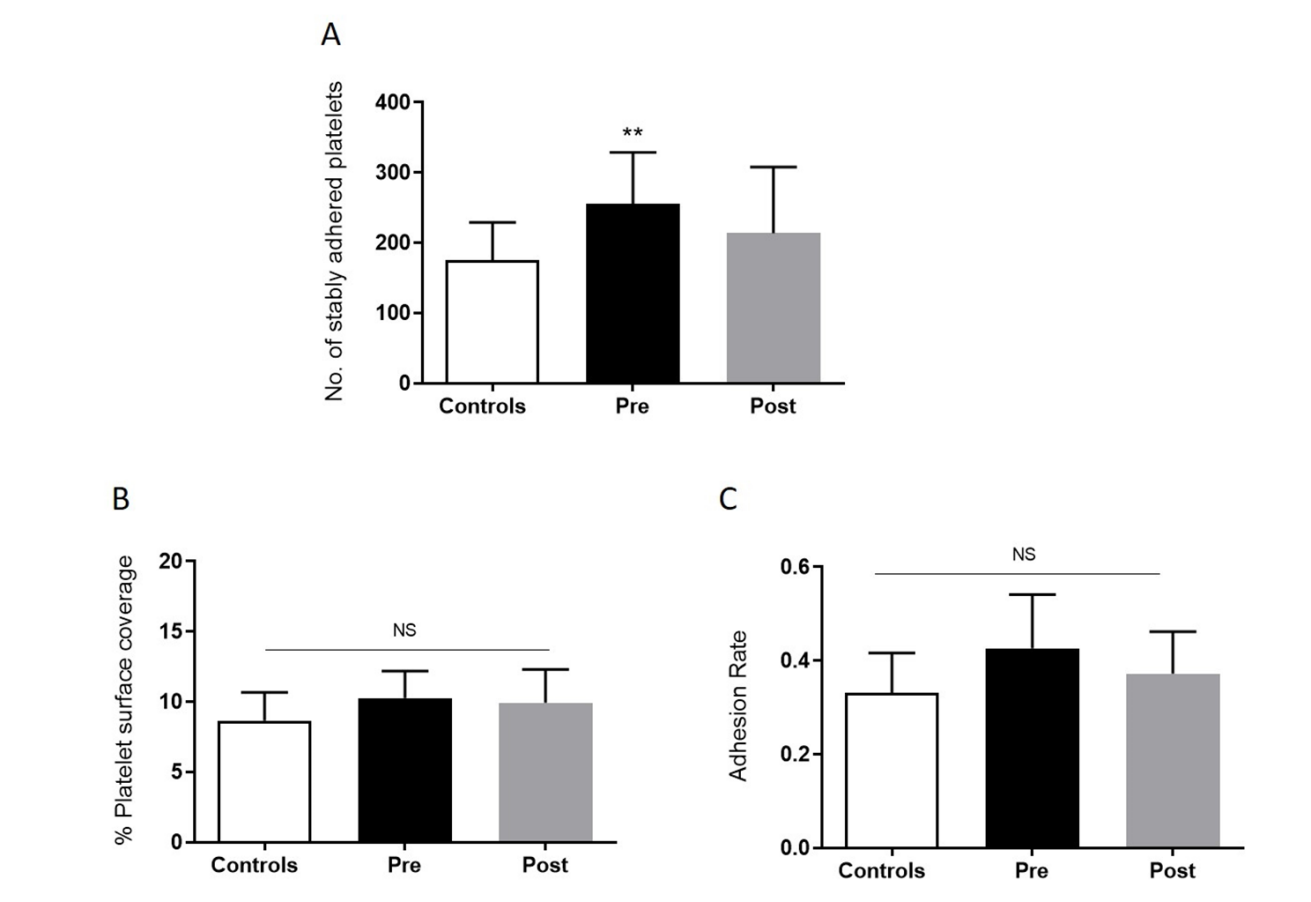
**Platelet –vWF adhesion in GHD pre and post GHT vs. healthy controls** The number of stably adhered platelets are significantly increased in GHD (n = 12) vs. controls, (n = 12). (**A**) Number of stably adhered platelets, (**B**) Percent surface coverage (**C**) adhesion rate measured using the dynamic platelet function assay. Data are represented as Mean ± SD (*p < 0.05, **p < 0.01, ***p < 0.001). All samples were measured in triplicate in the DPFA

Platelet adhesion as measured by the number of stably adhered platelets (static platelets) was also found to be significantly elevated in GHD pre therapy vs. controls, (p = 0.0087), with a reduction in levels observed post GHT, Fig. 3A. Percent surface coverage and adhesion rate was not significantly changed, Fig. 3B C. Average values of all platelet behavioural outputs measured for GHD pre and post GHT vs. healthy controls can be seen in Table 3. Of note, platelet counts for both GHD and healthy control groups were not significantly different, p = 0.96. No significant differences were observed in repeat DPFA tests on the control cohort performed on different occasions.

**Table 3:** Measurements of platelet behavioural parameters in the DPFA in healthy controls compared to GHD pre and post GHT.

Parameter	Healthy Control (n = 12)	Pre GH therapy (n = 12)	Post GH therapy (n = 12)	P value (ANOVA)
**Platelet Tethering**				
- Tracks (n)	356 ± 42	634 ± 86	621 ± 83	0.04*
**Platelet Rolling**				
- Translocation Distance (µm) - Translocation speed (µm/s) - Translocating Platelets (n)	13.05 ± 1.6 7.45 ± 1.3 248 ± 32	13.14 ± 0.7 8.16 ± 1 449 ± 63	16.99 ± 0.9 10.63 ± 0.89 460 ± 59	0.01* 0.03* 0.009**
**Platelet Adhesion**				
- Static platelets (n) - Adhesion Rate - % Platelet Surface Coverage	175 ± 16 0.33 ± 0.02 8.67 ± 0.58	256 ± 21 0.43 ± 0.03 10.28 ± 0.55	214 ± 27 0.37 ± 0.03 9.9 ± 0.69	0.04* 0.14 0.11

Results are expressed as the mean ± SD and were compared by one way ANOVA for GHD cohorts, pre and post GHT vs. healthy controls. Values were considered statistically significant where p < 0.05*, < 0.01**

## Discussion

In this paper we present the first report to our knowledge on altered platelet dynamics measured under arterial flow conditions in GHD pre vs. post GH replacement therapy and in comparison to a healthy control cohort. The data show differences in distinct stages of platelet-vWF interactions mediated by GPIBα as measured by seven previously validated behavioural outputs of platelet function in our assay [28]. We have shown an altered baseline of platelet function in GHD pre therapy vs. healthy controls which appeared to revert towards the control phenotype post GHT. Specifically, there were significant increases in the numbers of platelet tracks, stably adhered (static platelets) accompanied by trends towards increases in adhesion rate and the percent surface coverage. Taken together, increased tethering and adhesion properties imply a propensity towards a more “sticky” platelet in GHD vs. healthy controls and may be suggestive of intrinsic platelet differences in GHD patients. These differences however may arise from other factors associated with hypopituitarism and are not necessarily due to growth hormone deficiency alone.

It should be noted that whilst differences between GHD and controls are representative of the majority of samples tested, changes in each parameter (i.e. reduced platelet-vWF associations) post GHT were confined to approximately seven subjects. The remainder, n = 5 (3 F; 2 M) surprisingly displayed an increase in platelet-vWF interactions post GHT. The reasons for this remain unclear, however we could speculate that GH replacement alone is not sufficient to negate platelet abnormalities and these increases in platelet-vWF interactions may represent additional cardiovascular risk to these individuals.

Interestingly, the outputs relating to the distance and speed at which the platelets roll across the vWF surface were similar between controls and GHD pre therapy individuals despite greater numbers of translocating platelets being seen. However, post GHT there was a marked increase in both parameters. These findings could complement previous research where a decrease in carotid artery intima-media thickness (IMT) following GH replacement resulting in an improvement of blood flow in the brachial artery has been reported [29]. Platelet activation markers were not measured in this study. However, we have previously modelled the kinetics of platelet adhesion *in silico* and via in vitro experiments using the DPFA assay to investigate inhibition of GP1B and GPIIBIIIa resulting in decreased platelet adherence parameters and increased platelet velocity [30]. Therefore increases in distance and speed parameters measured in this study may reflect reduced signaling within platelets, and consequently slower activation of receptors that mediate stable adhesion.

It is well documented that GHD affects the cardiovascular system in several ways and likewise GH replacement therapy has been shown to exert sustained beneficial effects on several cardiovascular risk factors. Studies have shown that GHD can be associated with impaired cardiac performance, reduced left ventricular mass and decreased exercise capacity [31]. Short-term and placebo-controlled studies have shown that GH replacement in adult GHD patients, has an anabolic effect on cardiac structure resulting in an improvement in both diastolic and systolic function [32, 33].

Increased risk of cardiovascular morbidity has also been reported in patients with acromegaly [34]. Investigations into platelet involvement in acromegaly report increased platelet activity and mean platelet volume which is not altered following medical intervention to control growth hormone hypersecretion [35]. The pattern of cardiovascular disease however in acromegaly is different from that of GH deficiency. Patients with acromegaly tend to develop specific acromegalic cardiomyopathy related to excess GH level, also acromegalic patients have a significantly increased risk of hypertension and diabetes which contribute to additional cardiovascular morbidity especially strokes. Obstructive sleep apnea is also a major risk factor for cardiac dysfunction in acromegaly.

There is a paucity of studies however relating to the cellular and molecular mechanisms underpinning the pathology of GHD associated increased risk of vascular disease. In particular there is a lack of work with regard to the role of platelets in this area. Reis et al., 2005 reported a type of platelet hyperreactivity in untreated GHD vs. control and GHT individuals as evidenced by increased thrombin evoked Ca2+, collagen and ADP induced platelet aggregation [36]. The GH treated group maintained levels of GH following withdrawal of treatment which may account for the similarities to the control cohort. The authors suggest the potential for the use of platelets as a predictor of cardiovascular risk in GHD. An earlier study by Besser et al., 1975, reported impaired platelet aggregation in healthy males treated with ‘GH release-inhibitory hormone’ (Somatostatin) [37]. Here we examined platelet aggregation for a number of patients in response to agonists arachidonic acid (AA), adenosine diphosphate (ADP), collagen, thrombin receptor activating peptide (TRAP) and epinephrine. This was not carried out for all patients pre and post GHT due to limitations in the amount of platelet rich plasma (PRP) collected and hence data was incomplete and not shown. Results were variable however in comparison to our control cohort we observed elevated, but non-significant ADP and Arachidonic acid induced platelet aggregation in the majority of GHD subjects which was also reduced post GHT. We did not examine the direct impact of GHT in vitro and to our knowledge this has not been elucidated in the literature. A recent study by Karolczak et al., has reported the impact of cortisol on platelet reactivity in non-GHD individuals between 60 and 65 years old where lower cortisol levels were associated with increased platelet aggregation with ADP and AA but not collagen and the reverse was observed with higher cortisol levels [38]. GH therapy has been reported to reduce cortisol metabolism [39] and can also affect circulating T4 and T3 levels so it is possible that platelet reactivity observed in this study could be attributed to indirect effects of growth hormone/IGF-1.

It is evident from the literature that platelet function/activity can be affected in many other chronic diseases associated with significant increased vascular risk. A prime example of this is in diabetes mellitus (DM). Platelets from patients with type 1 and type 2 diabetes exhibit enhanced platelet aggregation activity early in the disease course that may precede the development of CVD [40]. This can be explained by several biochemical abnormalities which result in platelet hyperreactivity such as increased expression of activation-dependent adhesion molecules, or the presence of activated forms of GpIIb-IIIa, lysosomal Gp53, thrombospondin, and P-selectin which ultimately lead to increased aggregability [41]. A study by Gremmel et al., demonstrated that hyperreactivity in platelet function can also be seen in chronic kidney disease (CKD), [42] and we have recently reported normalized platelet-vWF interactions post successful kidney transplant using our DPFA assay [27].

With respect to the reported gender based risk of vascular disease in GHD it has been shown that women remain at a higher risk [1, 2] We did not uncover any discernable changes between males and females in our GHD cohort however, a larger group would need to be examined to draw any robust conclusions on this topic. There were no new other medications that subjects took regularly that could impact on platelet function or indeed interact with GHT. One subject on daily Aspirin was removed from the platelet analysis. In addition, other pituitary replacement therapies were optimised and did not change significantly after GHT. WCC was higher in the GHD group compared to the controls and also decreased post GHT. The significance of this is uncertain. A study by Bergamaschi et al., investigated the effect of GHT on blood cell counts in GHD children and adults [43]. This study found no effect of GHT on WCC and platelet count, however, subjects in the study who had normochromic normocytic anaemia, had restoration of their haemaglobin levels to normal range. Prothrombin time (INR) was significantly higher in the control group compared to the GHD group but changes in INR were not observed post GHT in GHD patients. Again, the reason for this is uncertain and it may be a result of the small sample size. We found no difference in platelet counts for both GHD and healthy control groups which is consistent with other studies [44].

The main limitation of our study is the small population size. Larger, prospective studies are required to further investigate our findings and to also assess whether these differences in platelet-vWF interactions observed, translate into markers of thrombotic tendencies.

In conclusion, our data demonstrate that GHD patients, pre–treatment, have altered baseline platelet profiles when compared to matched healthy controls. The differences observed in platelet–vWF interactions between these two groups appear to be suggestive of platelets being pro–thrombotic in GHD, with increased adherence properties. GH replacement led to reduced platelet-vWF interactions which arguably may result in platelets being less likely to adhere and thus less likely to thrombose when compared to the pre–treatment GHD state. Our findings could represent yet another pathological process contributing towards the increase in vascular disease seen in GHD hypopituitary patients.

## Data Availability

The datasets used and/or analysed during the current study available from the corresponding author on reasonable request.
